# A critical review on experimental *Streptococcus* *suis* infection in pigs with a focus on clinical monitoring and refinement strategies

**DOI:** 10.1186/s12917-023-03735-9

**Published:** 2023-10-05

**Authors:** Carolin Liedel, Karoline Rieckmann, Christoph G. Baums

**Affiliations:** Institute of Bacteriology and Mycology, Centre for Infectious Diseases, Faculty of Veterinary Medicine, Leipzig University, An den Tierkliniken 29, Leipzig, 04103 Germany

**Keywords:** *Streptococcus suis*, Pig, Experimental infection, Refinement, Scoring, Humane endpoint

## Abstract

*Streptococcus suis (S. suis)* is a major pig pathogen worldwide with zoonotic potential. Though different research groups have contributed to a better understanding of the pathogenesis of *S.* *suis* infections in recent years, there are still numerous neglected research topics requiring animal infection trials. Of note, animal experiments are crucial to develop a cross-protective vaccine which is highly needed in the field. Due to the severe clinical signs associated with *S.* *suis* pathologies such as meningitis and arthritis, implementation of refinement is very important to reduce pain and distress of experimentally infected pigs. This review highlights the great diversity of clinical signs and courses of disease after experimental *S.* *suis* pig infections. We review clinical read out parameters and refinement strategies in experimental *S.* *suis* pig infections published between 2000 and 2021. Currently, substantial differences exist in describing clinical monitoring and humane endpoints. Most of the reviewed studies set the body temperature threshold of fever as high as 40.5°C. Monitoring intervals vary mainly between daily, twice a day and three times a day. Only a few studies apply scoring systems. Published scoring systems are inconsistent in their inclusion of parameters such as body temperature, feeding behavior, and respiratory signs. Locomotion and central nervous system signs are more common clinical scoring parameters in different studies by various research groups. As the heterogenicity in clinical monitoring limits the comparability between studies we hope to initiate a discussion with this review leading to an agreement on clinical read out parameters and monitoring intervals among *S. suis* research groups.

**Supplementary Information:**

The online version contains supplementary material available at 10.1186/s12917-023-03735-9.

## Background

In 1959 the 3R principles (*replacement, reduction, refinement*) were initially proposed by Russel and Burch [1]. They wanted to achieve better treatment of laboratory animals and to improve scientific quality [2]. In recent years it has become increasingly important to reduce the number of animals used in research and to alleviate their harm and pain by applying the 3R principles [3, 4]. However, some pathogenesis and immunogenicity studies require the use of animal experiments due to the complexity of host–pathogen interactions and the host immune system [5]. Examples in *S. suis* research are loss-of-function studies [6] designed to read out the role of a putative virulence factor in causing meningitis and vaccination studies reading out adaptive immunity [7]. Researchers have to follow international rules and guidelines for animal experiments including 3R principles [3] to reduce pain and distress of animals where possible without jeopardizing the scientific validity [8]. Detailed reporting on animal research following e.g. the ARRIVE Guidelines ensures reproducibility and maximal research output which helps to reduce animal experiments in the future [9].

*S. suis* is a major porcine pathogen in the pig industry worldwide. It is a very diverse pathogen with 29 known serotypes [10–12]. There are important differences between countries regarding the prevalence of major serotypes with serotype 2 as the most prevalent causing disease in pigs worldwide [13]. *S.* *suis* can colonize the upper respiratory tract of pigs without causing disease [14]. However, invasive strains induce severe clinical signs of disease due to meningitis, arthritis, septicemia and endocarditis and may also lead to acute death. As such, infections with *S. suis* can cause high economic losses in pig husbandry due to mortality, lower weight gain and costs for treatment and prevention [11, 15]. *S.* *suis* is also a relevant human pathogen causing mainly meningitis, the streptococcal toxic shock-like syndrome and septicemia, especially in Asian countries [16, 17]. Consumption of raw pork or blood of pigs suffering from *S.* *suis* disease is a known risk factor for this zoonosis [17]. Moreover, humans may also be infected through wounds [17, 18]. Although knowledge on *S.* *suis* has improved in the past years [19], there are still important research questions regarding pathogenesis as well as development of a cross-protective vaccine that require experimental work with animals. So far there is no effective cross-protective commercial vaccine available [20] and autogenous vaccines as well as antibiotics are used to control *S.* *suis* diseases [15, 19].

The 3R´s include reduction of animal experiments [2]. This can be achieved through different measures which also play an important role in experimental *S.* *suis* pig infections. On the one hand, careful biometrical planning and selection of an appropriate study design might be used to reduce the number of animals [4, 8]. Furthermore, different *S.* *suis* research groups have conducted in vitro tests to read out host–pathogen interactions and immune responses which goes along with replacement of experimental infections in accordance with the 3R´s. Examples are serum or blood survival assays [21–24], opsonophagocytosis assays [7, 25–27] and cell culture models [28, 29]. Specifically, comparison of survival of wild type and isogenic mutants in blood e*x vivo* generates data on putative virulence factors involved in immune evasion mechanisms crucial for bacteremia [25, 30].

Arthritis, meningitis and other pathologies induced through experimental *S.* *suis* infection are associated with pain-related distress [31]. Therefore, adequate refinement is crucial to reduce distress in infected pigs. This review on 68 experimental *S.* *suis* infection studies in pigs, published between 2000—2021, focuses on refinement strategies, e.g. housing and handling, clinical monitoring and scoring and humane endpoints applied to minimize the burden of experimentally infected pigs. It highlights differences in clinical monitoring, read out parameters as well as humane endpoints applied by different *S.* *suis* researchers.

## Methods

The review includes 68 articles dealing with experimental *S.* *suis* infection in pigs published between January 1, 2000 and December 31, 2021. The following keywords were used in different combinations for article search using NCBI and Google scholar database: *Streptococcus* *suis*, experimental infection, challenge, vaccination, pig and swine. A few articles were found through references in other publications on NCBI or Google scholar database. Only articles were included in which clinical monitoring after experimental infection of pigs was performed due to potential development of signs typical for *S.* *suis* disease. We excluded studies written in a different language but English, studies dealing only with experimental infection of mice or natural *S.* *suis* infection, studies with permanent anesthesia after experimental infection, case reports and co-infection studies with the primary aim to read out clinical signs of another pathogen than *S.* *suis*. Furthermore, two studies were not considered if data suggested that experimental infection was not successful, e.g. the study by Warneboldt et al*.* (2016) describing oral application of *S.* *suis* [32].

## Results

### Status of piglets and experimental design of *S*. *suis* infection

**Fig. 1:**
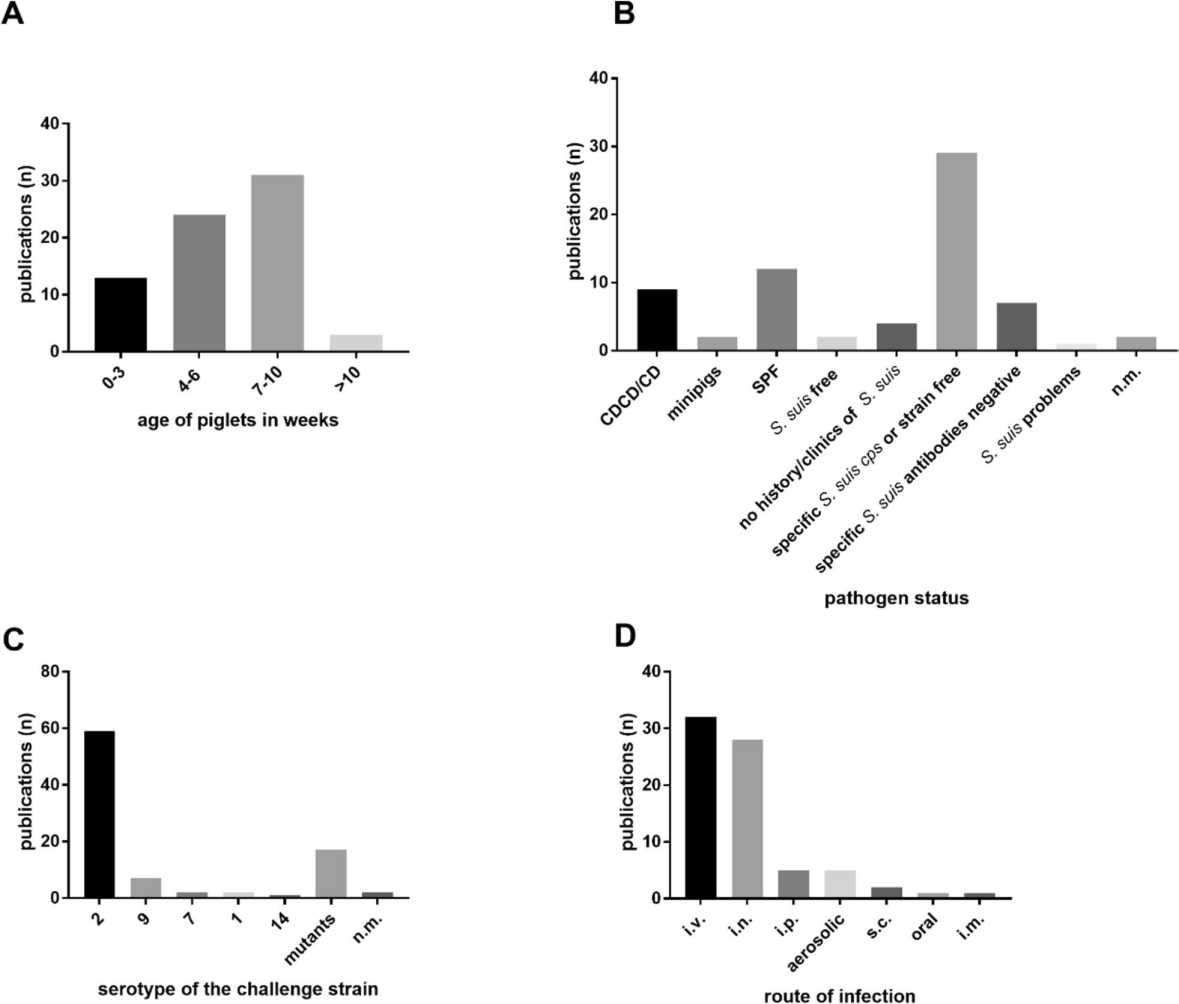
Age (**A**), infection status (**B**), *S.* *suis* serotype (**C**) and application route (**D**) in the reviewed publications on experimental pig infections (*n* = 68). In case of pigs of various ages (**A**), different serotypes (*cps*) (**C**) or infection routes (**D**) were used within one published study, it is listed under all respective categories. All mutants used in the reviewed studies were generated from serotype 2 wildtype strains. **C**. The pathogen status refers to the *S.* *suis* status of pigs (**B**). Pigs with a different immunity status e.g. caesarean-derived and colostrum-deprived (CDCD) pigs, that lack maternal immunity, as well as conventional pigs with potential background immunity were used. In some publications the *S.* *suis* pathogen status (*n* = 2/68) or serotypes (*n* = 2/68) of the challenge strain was not mentioned (n.m.)

Sixty-eight studies published between 2000 and 2021 including experimental *S.* *suis* infections in pigs were reviewed. We cannot exclude that the restriction to this period and the parameters used to identify publications are associated with a bias, as there are numerous older studies describing experimental infection of pigs with *S. suis*. However, information on refinement measures such as defined humane endpoints are generally scarce in older publications. Furthermore, clinical scores were only published more recently. Of the reviewed articles, most studies focused on pathogenesis (*n* = 41/68) or development of vaccines (*n* = 27/68) against *S.* *suis* disease in pigs. Throughout the period under review there were only a few publications dealing with *S.* *suis* coinfections (*n* = 9/68). These publications included infection models with piglets experimentally infected with porcine respiratory and reproductive syndrome virus (PRRSV) [33–37] (*n* = 5/9), *Bordetella bronchiseptica* [38, 39] (*n* = 2/9), porcine circovirus type 2 [40] or swine influenza virus [41] (each *n* = 1/9), followed by *S.* *suis* infection. Coinfection was used as a predisposing factor to promote the clinical manifestation of *S. suis* infection. In the reviewed studies mainly four- to six- (*n* = 24/68) and seven- to ten- (*n* = 31/68) week-old piglets were used for experimental infection with *S.* *suis* (Fig. 1A). This covers the period of time with the highest risk for pigs to get affected by *S.* *suis* disease in the field [11]. In particular most piglets develop signs of *S.* *suis* disease after weaning in association with different stressors [42]. For this, some studies have targeted weaners for experimental *S.* *suis* infection [43–47]. Unfortunately, many studies did not specify the duration between weaning and experimental infection. Even zero- to three-week-old piglets were used in a few studies (*n* = 13/68), whereas piglets older than ten weeks were rarely included (*n* = 3/68) (Fig. 1A). Of note, only a minority of experiments were conducted with piglets that lack maternal immunity, e.g. cesarean-derived and colostrum-deprived (CDCD) or just colostrum-deprived (CD) piglets (*n* = 9/68). More often conventional pigs regarded to be free from specific *S.* *suis* serotypes or strains (*n* = 29/68) were used (Fig. 1B). If described, the *S.* *suis* negative status for specific serotypes of the pigs was either determined by measurement of serum antibodies [6, 34, 41, 48, 49] or microbial screening of tonsil tissue or tonsil and nasal swabs e.g. through PCR [24, 26, 43, 47, 50–56]. The investigations on the *S.* *suis* status refer either exclusively to the animals used in the experiment [26, 34, 41, 43, 50, 52, 53, 57] or to the screening of the original herd over several years [24, 37, 51, 54]. However, defining piglets to be free from specific serotypes or antibodies does not exclude a substantial level of background immunity. Since nearly all pigs within a herd are colonized with *S.* *suis* in the upper respiratory tract, specific or unspecific cross-reactive antibodies are detectable in serum of these pigs [14, 58]. Some publications describe the usage of specific-pathogen-free (SPF) pigs (*n* = 12/68) (Fig. 1B). However, this does not mean that pigs were generally free from *S.* *suis* [59]. The term SPF-pigs is used in the sense that the animals are free from specific *S.* *suis* serotypes not *S.* *suis* in general [23, 47, 54, 56, 60]. Nevertheless, other studies use the term SPF without further clarification [46, 61–68]

Worldwide, the most prevalent serotype leading to disease in pigs and humans is *S.* *suis* serotype 2 [12, 13]. Accordingly, this was also by far the most commonly administered serotype in experimental *S.* *suis* pig infections (*n* = 59/68) (Fig. 1C), followed by *S.* *suis* serotype 9 (*n* = 7/68) (Fig. 1C). Serotypes rarely used in experimental *S.* *suis* infections in pigs were serotype 7 (*n* = 2/68), serotype 1 (*n* = 2/68) and serotype 14 (*n* = 1/68). Consequently, there is currently little data on experimental pig infections with other serotypes than serotype 2 as well as infection in pigs younger or older than four—ten weeks.

Pallarés et al. (2003) [47] conducted an experimental study comparing different routes of application. The authors recommended an intranasal model including a predisposition after local application of acidic acid as this model mimics the natural route of exposure and is effective in induction of typical pathologies. In the reviewed studies, intranasal (i.n.) (*n* = 28/68) or aerosolic (*n* = 5/68) applications were less frequently used than the intravenous (i.v.) route (*n* = 32/68). One limitation of intravenous models is the lack of host–pathogen interactions on mucosal surfaces though they are considered the initial steps in the pathogenesis of *S. suis* disease [14]. Noteworthy, intranasal application of a serotype 9 strain did not result in clinical signs of disease in contrast to i.v. application [69]. This suggests that induction of disease after intranasal application only works with a limited number of strains, at least in conventional piglets. The intraperitoneal (i.p.) route of infection is used infrequently in pigs (*n* = 5/68), in contrast to murine *S.* *suis* models [59]. Sporadically, subcutaneous (s.c.) (*n* = 2/68), oral (*n* = 1/68) or intramuscular (i.m.) (*n* = 1/68) infections were conducted (Fig. 1D). Among the reviewed experimental infections of pigs with *S.* *suis* there is substantial variation in i) the source and infection status of the animals, ii) the age group, iii) the challenge strain and iv) the application route.

### Housing and handling of piglets

Adequate housing and handling are fundamental requirements in animal experiments which otherwise may strongly contribute to distress of animals. As claimed by the Directive 2010/63 EU the facilities have to provide an environment covering the physiological and ethological needs of the species [70]. The EU-Directive requires social housing of animals and species specific environmental enrichment to reduce stress-induced behavior [70]. Regarding pigs, species specific environmental enrichment includes chewable and deformable materials, most suitable straw, which can be explored and moved [71]. As the pigs usually lose interest in materials within a few days, they have to be exchanged regularly [71]. To the best of our knowledge, it has not been investigated if environmental enrichments make a difference for transmission of *S. suis*. In studies designed to read out transmission [72, 73], such a link should be considered, even more as *S. suis* is commonly present in porcine saliva [74]. As mentioned in the ARRIVE Guidelines housing and husbandry conditions as well as acclimatization periods have an impact on well-being of animals and research outcome [9]. Therefore, it is relevant to report conditions which might influence study outcomes. Nevertheless, housing conditions (e.g. housing system, climate conditions, food and water supply, biosecurity level, group composition, environmental enrichment) are rarely described in research articles on *S.* *suis* pig infections. Usually, only water supply and feeding are specified [33, 34, 43, 45, 47, 53, 56, 60, 72, 75–79]. Nevertheless, almost all articles mention approval of their animal experiments by an ethical/local committee and/or ethical guidelines or laws in force for the use of laboratory animals (*n* = 58/68). This suggest that animals were housed under adequate conditions even if housing is not described in detail.

After the piglets have been moved to the experimental facilities, an acclimatization may further reduce stress of piglets induced through transport and the new environment [80]. This was described in a few articles (*n* = 14/86) in which the time for acclimatization ranges from two to 18 days [34, 36, 40, 44, 50, 53, 54, 65, 75, 78, 81–84]. Acclimatization is important to eliminate changes in physiological parameters e.g. heart rate, cortisol levels and reduced feed intake caused through transport-associated stress and the new environment [80, 85]. Moreover, in the acclimatization period piglets should get used to examinations by animal caretakers and veterinarians such as measurement of inner body temperature and palpation of joints [85, 86]. Of note, piglets can be trained with a clicker and sweets to accept standing on a scale or application of drugs [85] to reduce handling stress. Training of pigs prevents fear related behavior which could otherwise distort study results [85]. However, certain stressful procedures like administration of the challenge strain or blood sampling from the V. cava cranialis are difficult to train. Nevertheless, positive conditioning by sweets after stressful procedures can reduce fear of piglets. The DIRECTIVE 2010/63/EU requests the use of anesthesia or analgesia for procedures in animal experiments to reduce distress whenever it is possible and not in contrast to the aim of the study [70]. Accordingly, it is advisable to conduct stressful procedures like experimental infection [7, 69, 72, 83, 87] and euthanasia [72, 75, 79, 87–89] under anesthesia.

### Monitoring of pain and distress in pigs

The recognition of pain and distress in pigs is important to evaluate refinement strategies and, importantly, to introduce further steps to alleviate it. Commonly pain in animals is defined as “an aversive sensory experience caused by actual or potential injury that elicits protective and vegetative reactions, results in learned behavior, and may modify species specific behavior” [90]. To assess pain and distress in pigs it is crucial to establish adequate monitoring strategies. As claimed in guidelines of the national research council of the United States and the Directive 2010/63 EU personnel involved in use of laboratory animals have to be educated and trained in (i) performing procedures, (ii) designing projects and procedures, (iii) taking care of animals and (iv) killing of animals [70, 91]. Moreover, they need profound knowledge of the anatomy, physiology and species specific behavior of pigs as well as experience in recording specific clinical signs of *S.* *suis* disease in pigs [91, 92]. As an example, it is important to clearly recognize opisthotonos and ataxia as leading clinical signs of meningitis [47]. Furthermore, isolated lying and reduced frequency of movements are putative unspecific signs of disease. It is crucial to predefine clinical recording and humane endpoints before the challenge experiment. In some countries like Germany it might be obligatory to draft a prospective severity assessment covering the whole period in which animals are used in the experiment [93]. Different approaches have been used to measure pain in pig experiments such as grimace scales [94, 95] or scoring systems including typical signs of pain, e.g. change in normal behavior or movement, pain vocalization and reduced feed intake [58, 96, 97]. These methods have to be well validated to surely measure pain and not behavior related to anxiety or fear [96]. In addition, physiological parameters like increased heart and respiratory rate and levels of stress hormone e.g. cortisol or glucose are related to stress and pain [96, 98, 99]. As recently shown blood cortisol levels are significantly elevated in piglets suffering from severe *S.* *suis* disease [58]. However, there are limitations due to the influence of the circadian rhythm and the handling of the animals on the cortisol level [100]. Different *S.* *suis* research teams have been using score sheets to define different levels of severity of the various clinical signs of *S.* *suis* disease (Table 1).

**Table 1:** Clinical scoring systems used in experimental *S.* *suis* pig infections (published between 2000 - 2021)

Study type	Experimental infection			Observation interval	Humane endpoint	Scoring criteria		Score	Reference
	Age of pigs in weeks	Sero-type	Route						
path.	4–5	2	i.n.	not defined	score > 2 on attitude or locomotion	body temperature	< 40.4°C	0	[43]
							40.5–40.9°C	1	
							41–41.4°C	2	
							41.5–41.9°C	3	
							> 42°C	4	
						attitude	normal attitude and response to stimuli	0	
							inactive and slow to respond with oculonasal secretions	1	
							only responsive to repeated stimuli	2	
							recumbent, nonresponsive, and unaware of surroundings	3	
							dead	4	
						locomotion	normal gait and posture	0	
							slight incoordination, lameness, and/or joint swelling but rises without assistance	1	
							clearly uncoordinated or lame but stands without assistance	2	
							severe lameness and/or severe ataxia	3	
							dead	4	
	4–5	2	i.n.	daily	see Li et al. 2007 [101]				[102]
	2–3	2	i.n.	daily	meningitis; recumbency due to lameness	respiratory disease see Halbur et al. 1995 [103]	normal-severe	0–6	[37]^c^
						CNS/swollen joints, lameness	normal	0	
							mild	1	
							moderate	2	
							severe	3	
vac.	6	2	i.n.	twice daily	severe CNS disease or lameness with recumbency	respiratory disease see Halbur et al. 1995 [103]	absence	0	[56]
							severe	6	
						lameness/CNS	absence	0	
							severe	3	
vac.	7	2	i.p.	3/day	score = 3 in either category and body temperature > 40°C for 2 consecutive days	behavior	normal attitude and response to stimuli	0	[75]
							slight depression with marginally delay in the response to the stimuli, but preserved appetite	1	
							moderate depression, animal only responds to repeated stimuli, reluctant to move, decreased appetite	2	
							severe depression, non-responsive, recumbent, incoordination, diminished appetite	3	
						locomotion	normal gait and posture	0	
							one joint affected, light lameness, but rises and moves without assistance	1	
							moderate lameness, 2–3 joints affected with the swelling but stands without assistance	2	
							severe lameness, ataxia 3–4 joints affected, recumbent and cannot stand or move	3	
						CNS	normal physiological behavior and response to stimuli	0	
							slight incoordination, strabismus	1	
							moderate incoordination, trembling	2	
							severe, lateral or dorsal head inclination, ataxia, opisthotonos, nystagmus, convulsions	3	
	8	2	i.v.	not defined	severe clinical signs (score > 4)	feed attitude	normal	0	[7]
							reduced	1	
							anorexia	2	
						behavior	normal	0	
							slightly depressed	1	
							depressed	2	
							moribund, does not stand up	3	
							dead	4	
						lameness	normal	0	
							lameness grade 1, pig avoids movement on the leg	1	
							lameness grade 2, pig does not stand on the leg	2	
						central nervous signs	normal	0	
							mild, signs as incoordination are only visible after manipulation of the pig	1	
							moderate, signs as incoordination, head tremor in rest	2	
							Severe, signs as nystagmus, opisthotonos, ataxia	3	
vac.	8–9	9	i.v.	8h	body temperature ≥ 40.5°C and apathy and anorexia over 32 h [51] /24 h [24]; polyarthritis; CNS dysfunction; score ≥ 25	body temperature	< 40°C	0	[24, 51]
	8	14	i.n.	8h			40.0 – 40.2°C	1	
							40.3 – 40.5°C	2	
							> 40.5°C	3	
						feed intake	good	0	
							moderate	1^a^	
							ceased	3^b^	
						lameness	no	0	
							low-grade	1	
							high-grade	3	
							recumbency, polyarthritis	25	
						behavior	fresh	0	
							faint	1	
							listless	10	
							central nervous disorders	25	
						respiratory signs	costo-abdominal	0	
							forced abdominal breathing	1	
							cyanosis (ears)	8	
	8	2	aerosol	not defined	see Okura et al. 2021 [43] except that only attitude and locomotion is scored				[101]
	11–12	2	i.p.	daily	animals unresponsive to stimuli; CNS; lameness score of 3	behavior	physiological	0	[26]
							depression	1	
							apathy	2	
						locomotion	physiological	0	
							slightly to moderately lame	1	
							severely lame/reluctant to stand	2	
							animal partially/completely down, i.e., animals can rise but lies down again within 10 s	3	
						CNS	absent	0	
							present	1	
	2–3	2	i.n.	daily	CNS disease; joint swelling, lameness with recumbency	see Thanawongnuwech et al.2000 [37]			[36]^c^

*path*. study on pathogenesis, *vac*. study on vaccination

^a^score of 2 in [24]

^b^score of 5 in [24]

^c^PRRSV and *S. suis* coinfection

**Fig. 2:**
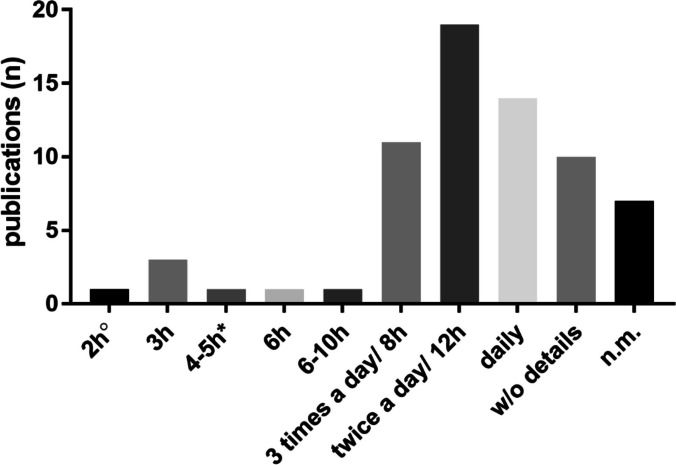
Monitoring intervals in the reviewed experimental *S.* *suis* pig infections published between 2000—2021 (*n* = 68). Several studies included clinical monitoring of the piglets but did not mention the interval (n.m.). ^O^2 hours (h) monitoring interval for 72 h. *4–5 h interval during the day, 8 h overnight

An adequate monitoring interval is crucial to detect clinical signs early and to reduce distress by euthanizing mortally ill piglets [104, 105]. In the reviewed publications, monitoring intervals varied substantially between different studies. Often piglets were monitored every 12 h (twice a day, *n* = 19/68) or on a daily basis (*n* = 14/68) (Fig. 2). Short fever peaks might be missed if the monitoring interval is longer than eight hours [51]. Furthermore, early signs of disease might rapidly progress to severe disease within eight hours [51]. In the case of longer monitoring intervals (≥ 12 h) *S. suis* infection might result in agony as piglets might not be euthanized after the onset of severe clinical signs. Importantly, this is not in accordance with the Directive 2010/63/EU as death as an endpoint has to be avoided [70]. Only a few publications use monitoring intervals below five hours (*n* = 5/68) (Fig. 2). Of note, one publication mentions a six hours interval with more frequent controls in the case of serious disease [45] as a measure to avoid animal suffering. However, if shorter clinical monitoring intervals are not applied to all piglets of the study, they might lead to a bias between two groups under comparison. If physical examinations are applied to all animals they might constitute an additional stressor to healthy piglets because the resting period is substantially reduced. Accordingly, researchers need to carefully weigh up the advantages and disadvantages of different monitoring procedures. Differences in chosen monitoring intervals of the reviewed publications can only partially be explained by different experimental settings which require more or less close monitoring due to the onset and severity of expected clinical signs of disease e.g. infection experiments with a highly virulent strain.

### Clinical scoring

**Fig. 3:**
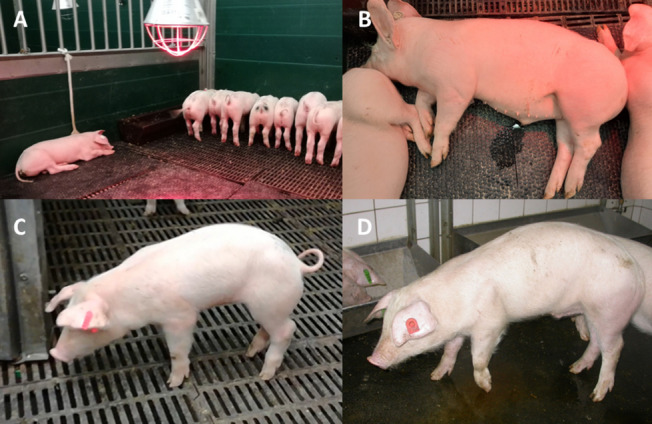
Examples of clinical signs of disease and distress in pigs after experimental *S.* *suis* infection. Experimental infection with *S.* *suis* induces very different clinics such as unspecific signs like reduced appetite (ability to stand up needs to be confirmed) (**A**), depressed and atypical behavior such as urination on the resting place (**B**), suggestive clinical signs such as kyphosis (in addition to a swollen tarsal joint) (**C**) as well as specific signs like a load lameness (**D**) or ataxia (see Video 1). Of note, many diseased piglets show only unspecific signs

Close clinical monitoring and pain assessment are crucial in animal infection experiments to introduce appropriate refinements to reduce distress and discomfort. As claimed by the ARRIVE Guidelines clinical monitoring should include general and study specific welfare parameters [9]. Thereby scoring systems, applicable by all trained persons examining and taking care of the experimental pigs, are a useful tool to assess welfare of the animals [105]. Additionally, they help to define criteria for euthanasia (humane endpoints) of piglets due to animal welfare reasons [105, 106]. The applied scoring system has to be adapted to the expected clinical signs [107] which depend on the animal species swine and the manifestation of *S.* *suis* infection. The application of a clinical scoring in experimental *S.* *suis* infections was only described in a few publications (*n* = 15/68). However, 11 *S.* *suis* publications with detailed information on clinical scoring are available [7, 24, 26, 36, 37, 43, 51, 56, 75, 101, 102]. They generally calculate a cumulative score for each animal or group (Table 1). Except for three studies [36, 37, 56], the scoring is the basis for predefined humane endpoints (Table 1). Signs of central nervous system (CNS) disorder and lameness as well as behavior are common parameters in applied score sheets. Respiratory signs, inner body temperature and feed intake are less often included as parameters. Respiratory signs were scored primarily in PRRSV and *S.* *suis* coinfection studies [36, 37]. Since *S.* *suis* is a porcine pathogen causing inflammatory disease, increase of inner body temperature is an important early indication for the onset of disease. Even though most publications include body temperature of pigs in their regular clinical monitoring (*n* = 60/68), only three published studies include the inner body temperature as read out parameter in their scoring system [24, 43, 51]. Reduced feed intake is an unspecific but clear indication of pain or distress in pigs [96]. Nevertheless, feed intake and appetite are parameters uncommonly recorded in experimental *S. suis* pig infections. Short peaks of elevated body temperature and reduced feed intake might be related to other factors but infection. For example, an increased inner body temperature is a common clinical sign in vaccinated animals [108] which can be accompanied with reduced feed intake. Nevertheless, monitoring of feed intake or appetite is in the authors` opinion of additional value in *S. suis* trials because many piglets show only unspecific signs of disease after experimental infection. Other parameters of applied clinical scoring systems are similar, but they differ considerably in their score points and their subdivision of the main parameters (Table 1). Furthermore, the gradation within a parameter varies substantially. For example, the parameter lameness/locomotion is subdivided into absent and severe in one study [56], whereas in another the same is subdivided into five grades [43]. More gradations within a parameter might help to highlight differences in severity of disease between individual piglets or relevant groups. However, grades have to be clearly defined so that they can be objectively applied by different researchers. Since score sheets are planned prior to the animal experiment based on the expected clinical signs, a re-evaluation after the experiment should be conducted [105]. We have revised our scoring system by increasing the score for moderate and ceased feed intake [24, 51]. This was done because ceased feed intake (> 24 h) is based on our experience a sign of severe disease in piglets experimentally infected with *S. suis*. We have observed moderate to severe cases of *S.* *suis* infection that are associated with temporarily increased body temperature, reduced feed intake (Fig. 3A), depressed and atypical behavior (Fig. 3B) or kyphosis (Fig. 3C). Specific clinical signs of *S.* *suis* infection like lameness (Fig. 3D) or ataxia (Video 1) were not recorded. Although these pigs did not show specific clinical signs of *S.* *suis* disease, they were euthanized if the described clinical signs continued longer than 24 h. This was done to prevent ongoing distress, suffering, pain and harm. An example for such a case is piglet #38 infected intravenously with *S.* *suis* serotype 9 within a vaccination trial. This piglet reached a maximum clinical score of 17 at one time point of clinical monitoring which was below the defined threshold score for immediate euthanasia of 25 [51]. However, piglet #38 cumulated score points at different clinical controls resulting in a cumulative score above 25 and thus euthanasia. Other piglets with the aforementioned moderate clinical signs showed convalescence within 24 to 32 h [51, 81]. Convalescence was associated with behavioral changes such as increased vigilance and movements, prominent feed intake and disappearance of fever. Vaccinated piglet #44, which was part of the same study as piglet #38, reached a maximum score of 15 one day post infection [51] but demonstrated convalescence within 24 to 32 h as described in the Results section [51] and as reflected by the curve of the rectal body temperature [51]. If piglets with moderate signs of disease such as #38 and #44 were euthanized immediately after the first signs such as elevated body temperature and ceased feed intake, such studies would lose sensitivity to detect partial protection. However, studies designed to read out other parameters such as transmission and colonization might implement other humane endpoints [72]. In accordance with official guidelines such as the Canadian Council on Animal Care “the earliest endpoint that is compatible with the scientific objectives of the approved protocol should be used” [109].

### Inner body temperature as a clinical read out parameter

After experimental infection with *S. suis*, an elevated inner body temperature is an important indicator of an early stage of disease [11]. A slight and often only very short increase in body temperature after experimental infection can be recorded in many infected piglets that do not develop other clinical signs of disease. As an example, an increase in body temperature up to 40.3°C was shown after i.n. experimental infection of pigs with an attenuated mutant of a serotype 2 strain [83]. An elevated body temperature up to 40.4°C was detected in a piglet experimentally challenged i.v. with *S.* *suis* serotype 9 suffering from endocarditis [81]. Experimental infection with *S.* *suis* serotype 9 and 2 might induce clinical signs of severe *S.* *suis* disease e.g. central nervous disorder accompanied with high fever up to 41.3°C [110] and 42.7°C [69]. For the definition of fever, the age of the pigs or method of measurement must be taken into account, since inner body temperature varies between different ages and methods [11, 111, 112]. Consequently, Baums et al. (2006) set different fever thresholds for weaners (≥ 41°C) and growers (≥ 40.5°C) [83]. In the reviewed articles fever thresholds for inner body temperature were mostly set above 40.0 or 40.5°C (*n* = 28/32) except for one study in which the temperature was measured in the ear and fever was defined as > 38.5°C (Table 2). In three studies, fever in pigs was set at > 41.0°C (Table 2). Although inner body temperature is commonly monitored in *S.* *suis* pig infections (*n* = 60/68), only a minority of publications clearly define a body temperature threshold of fever in pigs (*n* = 32/68) or include this parameter in the scoring system (Table 1). Humane endpoints generally do not refer to the inner body temperature (Table 3). In contrast, definitions of morbidity often include inner body temperature as an obligatory criterion [24, 25, 30, 51, 54, 87, 113]. Most of the reviewed articles do not specify the method of measuring inner body temperature (*n* = 48/60). In the other publications mainly recording of the rectal body temperature is described (*n* = 11/60), most likely as it is considered to be the “gold standard” for the core temperature [112, 114]. Since it is known that stress can increase body temperature it is important that pigs get used to rectal measurement through positive conditioning and training [85, 115]. This allows rectal body temperature to be recorded without restraining the animals. Alternatively, as reviewed by Schmid et al*.* (2021), subcutaneously implanted thermo sensors or non-invasive contact sensors were used in animals to measure core temperature [112]. Contactless infrared thermometry is less invasive, but it reflects body surface temperature which can be affected by environmental factors. Nevertheless, good correlations between infrared thermometry and rectal temperature have been described, especially for ear and eye [78, 100, 115]. Comparative evaluation of different methods of body temperature measurements has however not been conducted for piglets experimentally infected with *S.* *suis*, in contrast to PRRSV infected pigs [116].

**Table 2:** Definitions of fever in experimental *S.* *suis* pig infections conducted in the years 2000—2021

Study type	Experimental infection			Body temperature threshold of fever	Reference
	Age of pigs in weeks	Serotype	Route		
model	6–7	2	i.n.	≥ 41°C	[34]^a^
	7	2	i.v.		[117]
path.	4–5	2	i.n.		[83]^b^
path.	4	2	i.n.	≥ 40.5°C	[57]
	4–5	2	i.n.		[43]^c^
	4–5	2	i.n.		[87]^d^
	5	7	i.v.		[23]^d^
	8	2	i.n.		[25]^d^
	5–6	2	i.n.		[41]^c^
	3	2	i.v.		[46]^c^
	7–8	2	i.n.		[83]
vac.	8	14	i.n.		[24]^d^
	8–9	9	i.v.		[51]^d^
	8–9	2, 7	i.v.		[48]
	7–8	2	i.n.		[30]^e^
	9	2	i.n.		[113]^d^
	9	9	i.v.		[81]^d^
	6, 8	2	i.v.		[44]^d^
	9	2, 9	i.n., i.v.		[82]^d^
	7–8	2, 9	i.v.		[54]^d^
	2–3	2	i.n.		[36]
model	1–2	2	i.n., i.v.	> 40.0°C	[47]
path.	6	2	s.c.		[78]^f^
	6	2	aerosolic		[55]^g^
	0–1	2	i.n.		[35]
	1	2	i.n., i.v.		[118]
	3–4	2	i.n.		[37]
vac.	5	1, 2	i.p.		[89]^f^
	8	2	i.v.		[67]
	4	2	s.c.		[60]
	8	2	i.v.		[68]
vac.	8–9	1	i.p.	> 38.5°C^h^	[77]

*model* study on establishment of an infection model, *path*. study on pathogenesis, *vac*. study on vaccination

^a^ > 41°C

^b^ ≥ 41°C only for weaners

^c^ > 40.5°C

^d^defined as high fever

^e^ ≥ 40.2°C defined as fever and ≥ 40.5°C as high fever

^f^ ≥ 40°C

^g^defined as elevated body temperature

^h^measured as ear temperature

**Table 3:** Predefined humane endpoints leading to euthanasia of pigs in experimental *S.* *suis* infections published between 2000—2021

Study type	Experimental infection			Humane endpoints	Reference
	Age of pigs in weeks	Serotype	Route		
path.	4–5	2	i.n.	score > 2 on attitude or locomotion	[43, 102]^a^
vac.	8	2	aerosol		[101]^a^
vac.	8	2	i.v.	severe clinical signs (score > 4)	[7]^a^
vac.	11–12	2	i.p.	animals unresponsive for stimuli; CNS dysfunction; lameness score of 3	[26]^a^
path.	5	2	i.n.	“high clinical scoring”	[52]^a^
vac.	7–8	2	aerosol		[119]^a^
vac.	7	2	i.p.	score = 3 in either category and a body temperature above 40°C for 2 consecutive days	[75]^a,b,c^
path.	5	7	i.v.	fever ≥ 40.5°C, apathy and anorexia (persisting over 24h [24] /32h [51]); acute polyarthritis; CNS dysfunction; score ≥ 25 [51, 24]	[23]^a^^,b,c^
vac.	8	14	i.n.		[24]^a,^^b,c^
	8–9	9	i.v.		[51] ^a,b,c^
path.	4–5	2	i.n.	high fever (≥ 40.5°C), apathy and anorexia persisting over 36h/24h [81] ; CNS dysfunction; acute polyarthritis	[87]^b,c^
	8	2	i.n.		[25]^b,c^
	9	2	i.n.		[30]^b,c^
vac.	7–8	2	i.n.		[113]^b,c^
	9	9	i.v.		[81]^b,c^
path.	0–1	2	i.n.	consumption < 75% or more of dispensed diet for four feedings in a row and lameness, fever (> 40°C), or CNS disease	[35]^b,c,d^
vac.	8–9	2, 7	i.v.	≥ 40.5°C, apathy and anorexia; polyarthritis; CNS disorder	[48]^b^
	6, 8	2	i.v.		[44]^b^
	9	2	i.n., i.v.		[82]^b^
	7–8	9	i.v.		[54]^b^
path.	7–8	2, 9	i.n., i.v.	high fever (≥ 41°C weaners; ≥ 40.5°C growers), apathy and anorexia	[70]^b^
	4–5/7–8	2	i.n.		[84]^b^
model	7	2	i.v.	fever (≥ 41°C) or CNS dysfunction	[117]^b^
model	9	2	oral	(severe) clinical signs/disease e.g. arthritis/lameness/swollen joints w/o recumbency; meningitis/central nervous signs	[53]
	5–6	2	i.n.		[34]*
	1–2	2	i.v., i.n.		[47]
	7	2	i.v.		[120]
path.	6	2	s.c.		[78]
	6	2	i.n.		[56]
	(0)-1	2	i.n.		[38, 39]^e^
	3	/	i.v.		[121]
	6-(7)	2	aerosolic		[55, 79, 88]
	3	2	i.v.		[62]
	3–4	2	i.n.		[37]^d^
	3	2	i.v.		[46]
vac.	9	2	i.n.		[122]
	4	2	s.c.		[60]
	2–3	2	i.n.		[36]^d^
	8	2	i.v.		[67, 68]
path.	4–5	2	i.v.	not clearly defined (“for animal welfare or ethical reasons”)	[6]
	8	2	i.v.		[65]
	1	2	i.v., i.n.		[118]
vac.	7	9	i.n.		[72]
	9	2	i.v.		[123]

*path*. study on pathogenesis, *vac*. study on vaccination, *model* study on establishment of an infection model

^a^scoring system included in humane endpoints (h.e.)

^b^fever included in h.e

^c^duration of burden included in h.e

^d^PRRSV and *S. suis* coinfection

^e^*Bordetella bronchiseptica* pre infection

### Humane endpoints

The European directive 2010/63 claims that “The methods selected should avoid, as far as possible, death as an end-point due to the severe suffering experienced during the period before death. Where possible, it should be substituted by more humane endpoints using clinical signs that determine the impending death, thereby allowing the animal to be killed without any further suffering “ [70]. Accordingly, it is crucial to define humane endpoints in *S.* *suis* infection experiments adapted to the specific study [124] and comply a good balance between humane termination of the experiment and the scientific concern [125]. In the reviewed articles, CNS disorder, signs of polyarthritis and recumbency are often defined as humane endpoints (Table 3). Only few research groups have used their applied scoring system as part of the predefined humane endpoints (*n* = 11/68) (Table 3). Criteria infrequently considered are fever (*n* = 17/68) and anorexia (*n* = 15/68) (Table 3). The latter might be associated with pain in pigs (Fig. 3A) [96, 97], accordingly including this criterion in the predefined humane endpoints helps to reduce pain in infected animals. Several studies provide only very brief information on humane endpoints (Table 3), such as the occurrence of typical signs of severe *S.* *suis* disease (*n* = 20/68) or euthanasia of piglets due to animal welfare or ethical reasons (*n* = 5/68). Further studies do not include details on applied humane endpoints (*n* = 3/68) [50, 57, 76] or did not mention them at all (*n* = 16/68) [16, 27, 33, 40, 41, 45, 49, 61, 63, 64, 66, 77, 84, 89, 126, 127]. *S.* *suis* infections may not only result in acute severe disease, but in some cases also in a more moderate course often associated with a delayed onset, continuous lameness or intermittent fever [81]. This may lead to a persistent moderate disturbed general condition over a longer period of time and thus an ongoing burden for the pigs. However, the duration of burden is included as criterion for humane endpoints only in a minority of reviewed articles (*n* = 10/68). For example, Obradovic et al*.* (2021) [75] euthanized pigs with a score of three and an inner body temperature over 40.0°C for two consecutive days [75] taking into account ongoing fever in the humane endpoints. Furthermore, Feng et al. (2001) [35] considers persistent reduced feed intake. Piglets that consumed less than 75% of the diet during four feedings or showed fever were humanely killed [35].We have conducted numerous studies euthanizing piglets showing high fever, apathy and anorexia over time periods ranging from 24 to 36 h [23–25, 30, 51, 69, 81, 83, 87, 113] (Table 3). Nevertheless, adequate humane endpoints depend on the aim of the study and the desired scientific output.

## Concluding remarks, outlook and recommendations

We reviewed 68 publications describing clinical monitoring and refinement measures in studies with experimental infection of pigs with *S. suis*. As we considered only publications after the year 2000, there is most likely a bias regarding all *S. suis* publications as reporting of monitoring and requirements for animal welfare measures changed over the years. We envision that new methods will be introduced into clinical surveillance in the future to improve scientific outcomes and allow for more timely detection of disease onset. As such monitoring of behavior using video-recording, measurement of core body temperature using infrared thermography (IRT) or microchip transponder might be considered [114, 128–131].

This review documents a high level of heterogenicity in the status of the piglets (e.g. age and infection status), the experimental infection itself (challenge strain, route of application) and the clinical monitoring (interval, read out parameters) as well as humane endpoints This heterogeneity may make comparison of results of experimental *S. suis* infections much more difficult. Only 15 of the reviewed articles included scoring systems for clinical monitoring. Published score sheets exhibit substantial variations in the included parameters (e.g. body temperature and feed uptake) and their gradation. We recommend to implement clinical score sheets in future experimental infections of pigs and to conduct systematic physical examinations of piglets including scoring of behavior, locomotion and measurement of body temperature every 8h after experimental infection. Ideally also appetite or even better feed intake is assessed. Humane endpoints should be defined clearly prior to the experimental study as this is a very important refinement in many *S. suis* trials. Although the published score sheets already provide a suitable basis, coordination between *S. suis* scientists on the read-out parameters used and their gradation should be an important goal of future exchange. Ideally, guidelines for experimental *S. suis* infections in pigs would be established to ensure a high degree of comparability and reproducibility of scientific results and refinement measures.

### Supplementary Information

**Additional file 1: Video S1.** Pig with ataxia after experimental infection with S. suis. A piglet experimentally infected i.n. with *S. suis* serotype 14 shows ataxia as a signs of central nervous disorder 7 days after challenge.

## Data Availability

The datasets analyzed during the current study are available from the corresponding author on reasonable request.

## Abbreviations

CDCD: Cesarean-derived and colostrum-deprived
CNS: Central nervous system
i.m.: Intramuscular
i.n.: Intranasal
i.p.: Intraperitoneal
i.v.: Intravenous
PRRSV: Porcine reproductive and respiratory syndrome virus
SPF: Specific pathogen-free
s.c.: Subcutaneous
